# Artificial Intelligence–Based Electrocardiographic Biomarker for Outcome Prediction in Patients With Acute Heart Failure: Prospective Cohort Study

**DOI:** 10.2196/52139

**Published:** 2024-07-03

**Authors:** Youngjin Cho, Minjae Yoon, Joonghee Kim, Ji Hyun Lee, Il-Young Oh, Chan Joo Lee, Seok-Min Kang, Dong-Ju Choi

**Affiliations:** 1 Division of Cardiology, Department of Internal Medicine Seoul National University Bundang Hospital Seoul National University College of Medicine Seongnam, Gyeonggi-do Republic of Korea; 2 ARPI Inc Seongnam, Gyeonggi-do Republic of Korea; 3 Department of Emergency Medicine Seoul National University Bundang Hospital Seongnam, Gyeonggi-do Republic of Korea; 4 Division of Cardiology, Department of Internal Medicine Severance Hospital Yonsei University College of Medicine Seoul Republic of Korea

**Keywords:** acute heart failure, electrocardiography, artificial intelligence, deep learning

## Abstract

**Background:**

Although several biomarkers exist for patients with heart failure (HF), their use in routine clinical practice is often constrained by high costs and limited availability.

**Objective:**

We examined the utility of an artificial intelligence (AI) algorithm that analyzes printed electrocardiograms (ECGs) for outcome prediction in patients with acute HF.

**Methods:**

We retrospectively analyzed prospectively collected data of patients with acute HF at two tertiary centers in Korea. Baseline ECGs were analyzed using a deep-learning system called Quantitative ECG (QCG), which was trained to detect several urgent clinical conditions, including shock, cardiac arrest, and reduced left ventricular ejection fraction (LVEF).

**Results:**

Among the 1254 patients enrolled, in-hospital cardiac death occurred in 53 (4.2%) patients, and the QCG score for critical events (QCG-Critical) was significantly higher in these patients than in survivors (mean 0.57, SD 0.23 vs mean 0.29, SD 0.20; *P*<.001). The QCG-Critical score was an independent predictor of in-hospital cardiac death after adjustment for age, sex, comorbidities, HF etiology/type, atrial fibrillation, and QRS widening (adjusted odds ratio [OR] 1.68, 95% CI 1.47-1.92 per 0.1 increase; *P*<.001), and remained a significant predictor after additional adjustments for echocardiographic LVEF and N-terminal prohormone of brain natriuretic peptide level (adjusted OR 1.59, 95% CI 1.36-1.87 per 0.1 increase; *P*<.001). During long-term follow-up, patients with higher QCG-Critical scores (>0.5) had higher mortality rates than those with low QCG-Critical scores (<0.25) (adjusted hazard ratio 2.69, 95% CI 2.14-3.38; *P*<.001).

**Conclusions:**

Predicting outcomes in patients with acute HF using the QCG-Critical score is feasible, indicating that this AI-based ECG score may be a novel biomarker for these patients.

**Trial Registration:**

ClinicalTrials.gov NCT01389843; https://clinicaltrials.gov/study/NCT01389843

## Introduction

Heart failure (HF) is a major global health problem affecting millions of people worldwide, leading to significant morbidity, mortality, and health care expenditure [1-3]. Although several valuable biomarkers such as N-terminal prohormone of brain natriuretic peptide (NT-proBNP) [4,5] and cardiac troponins [6] have been introduced for patients with HF, their use in routine clinical practice is often constrained by their cost and limited availability.

Electrocardiogram (ECG) is an essential and cost-effective tool for evaluating cardiovascular diseases. ECG is widely available, noninvasive, and provides real-time information about cardiac electrical activity, which is crucial for detecting arrhythmias, ischemia, and other cardiac abnormalities. With advances in artificial intelligence (AI) and deep learning, there has been growing interest in employing AI algorithms to analyze ECG data and predict outcomes in patients with various cardiovascular conditions [7,8].

In this study, we investigated the utility of an AI algorithm that analyzes printed ECG images for outcome prediction in patients with acute HF. These findings will demonstrate the potential of AI-assisted ECG analysis for predicting outcomes in these patients, potentially overcoming the cost and availability constraints of current biomarkers.

## Methods

### Study Population

This was a substudy of the prospective multicenter Korean Acute Heart Failure (KorAHF) registry, which enrolled 5625 consecutive patients upon initial hospital admission for acute HF at 10 tertiary university hospitals in Korea. Details on the KorAHF registry objectives, design, and population are available on the clinical trial registration site (ClinicalTrials.gov NCT01389843) and have been published previously [9,10]. Briefly, patients who had signs or symptoms of HF and met one of the following criteria were eligible for enrollment in the KorAHF registry: (1) lung congestion or (2) objective left ventricular systolic dysfunction or structural heart disease findings. There were no exclusion criteria.

**Figure 1:**
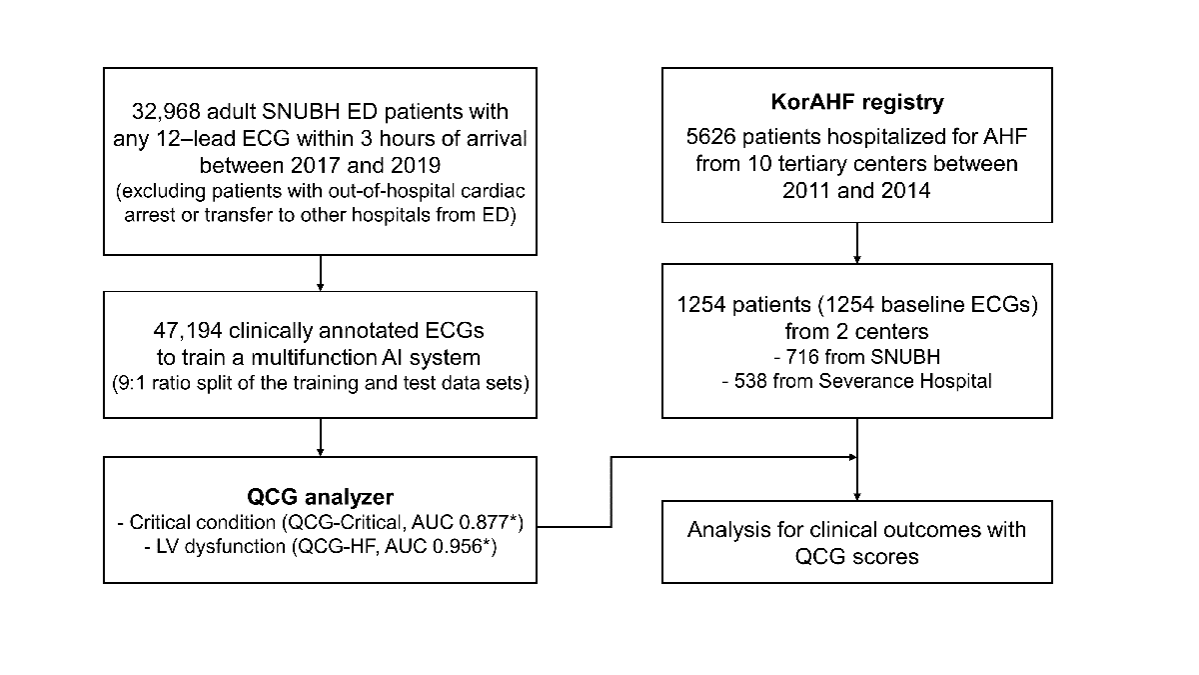
Flowchart of the training and validation study populations. The AI-ECG analyzer, named Quantitative ECG (QCG), was developed using 47,194 annotated ECG images of over 32,968 patients who visited the emergency department of SNUBH between 2017 and 2019. The QCG analyzer was applied to ECGs from a subpopulation of the KorAHF registry including patients with AHF enrolled between 2011 and 2014. *Internal validation results for two QCG scores. AHF: acute heart failure; AI: artificial intelligence; AUC: area under the curve; ECG: electrocardiogram; ED: emergency department; KorAHF: Korean Acute Heart Failure; LV: left ventricular; SNUBH: Seoul National University Bundang Hospital.

In this study, we retrospectively analyzed the prospectively collected data from 1254 patients who were hospitalized for acute HF from March 2011 to February 2014 at 2 out of 10 participating tertiary centers (Seoul National University Bundang Hospital and Severance Hospital) using the KorAHF registry (Figure 1). Additional ECG image data were collected for this study.

### Ethical Considerations

This study conformed with the principles outlined in the Declaration of Helsinki. The study protocol was approved by the institutional review board at Seoul National University Bundang Hospital (No. B-1104-125-014) and Severance Hospital (No. 2022-2166-001). The need for written informed consent was waived by the institutional review board. Our research strictly adheres to the Guidelines for Developing and Reporting Machine Learning Predictive Models in Biomedical Research [11].

### Clinical Follow-Up and Endpoints

Data collection methods have been previously described [9]. Briefly, data on patients’ clinical manifestations, biochemical parameters, medication, and outcome were collected using a web-based case report form for up to 60 months by research nurses. Outcome data on patients lost to follow-up were additionally collected from national death records.

The primary endpoint of this study was all-cause mortality. Secondary outcomes included in-hospital outcomes, especially in-hospital mortality. All deaths were considered to be cardiac-related unless a definite noncardiac cause could be established. All outcome data reported from the participating centers were reviewed by an independent clinical event adjudicating committee.

### AI Algorithm

Quantitative ECG (QCG) is an AI analyzer composed of an encoder part and multiple task-specific networks. The encoder part is a modified convolutional neural network with residual connections, squeeze excitation modules, and a nonlocal block. The task-specific networks are multilayer percetron models. The encoder part accepts 2D ECG images as input to produce a common numerical feature vector for downstream tasks. The encoder part was pretrained on 49,731 open ECGs using self-supervised learning schemes and then fine-tuned on 47,194 annotated ECG images of over 32,968 patients who visited the Emergency Department of Seoul National University Bundang Hospital between 2017 and 2019 using multitask learning schemes. The tasks include the classification of 12 rhythms (with 35 subtypes) and production of 10 digital biomarkers correlated with the risk of (1) being critically ill (shock, respiratory failure, or cardiac arrest), (2) cardiac ischemia (acute coronary syndrome, ST-elevation myocardial infarction, or myocardial injury as defined by an elevated troponin level), (3) cardiac dysfunction (pulmonary edema, left and right heart dysfunction, pulmonary hypertension, and clinically significant pericardial effusion), and (4) hyperkalemia. Several validation studies of the system have been published previously [12-14]. The collection of these AI algorithms has been developed into a mobile app (ECG Buddy, ARPI), which has been approved by the Korean Ministry of Food and Drug Safety.

In this study, two QCG features were evaluated: QCG-Critical for critical conditions such as shock or mortality and QCG-HF for a reduced echocardiographic left ventricular ejection fraction (LVEF) of <40%. The QCG scores, representing probability, ranged from 0 to 1.0, with 0 indicating low and 1.0 indicating high probability. With a 9:1 ratio split of the training and test data sets, the internal validation results for these two QCG features showed an area under the curve (AUC) of 0.877 for QCG-Critical and 0.956 for QCG-HF. The composition of the training and validation data sets is presented as a flowchart in Figure 1.

### Statistical Analysis

Categorical variables are reported as frequencies (percentages) and continuous variables are expressed as means (SD) or medians (IQR). The two key AI-driven scores (QCG-Critical and QCG-HF) were analyzed as continuous variables. The Student *t* test and *χ*^2^ (or Fisher exact) test were used to compare the baseline clinical characteristics between the two groups. The discrimination performance of QCG scores for in-hospital outcomes was evaluated using receiver operating characteristic (ROC) curve analysis. The AUC values were compared using the DeLong test. The logistic regression model was used to estimate the odds ratios (ORs) and 95% CIs. Survival analysis was performed using the Kaplan-Meier method, and the Cox proportional hazard model was used to estimate the hazard ratios (HRs) and 95% CIs for the clinical outcomes. Multivariable analysis was performed with the inclusion of clinically relevant variables.

All tests were two-tailed and a *P* value <.05 was considered statistically significant. Statistical analyses were performed using R programming version 4.3.0 (The R Foundation for Statistical Computing).

## Results

### Baseline Characteristics

Data of 1254 patients (716 from Seoul National University Bundang Hospital and 538 from Severance Hospital) were analyzed. Among the 1254 patients, 53 (4.2%) experienced in-hospital cardiac death. The baseline characteristics of the study population according to the in-hospital outcomes are shown in Table 1. Compared with survivors, patients who died in the hospital were older, had a higher prevalence of ischemic heart disease, lower LVEF, and higher NT-proBNP levels. By contrast, atrial fibrillation (AF) was more frequent in survivors. The QCG-Critical and QCG-HF scores were significantly higher in patients who experienced in-hospital cardiac death than in survivors (*P*<.001) (Table 1 and Figure S1 in Multimedia Appendix 1).

**Table 1:** Baseline characteristics.

Characteristics		Total (n=1254)	In-hospital cardiac death (n=53)	Survivors (n=1201)	*P* value
Age (years), mean (SD)		69.8 (14.7)	74.0 (14.5)	69.6 (14.1)	.03
Male, n (%)		673 (53.7)	29 (54.7)	644 (53.6)	.99
Hypertension, n (%)		843 (67.2)	31 (58.5)	812 (67.6)	.22
Diabetes mellitus, n (%)		499 (39.8)	23 (43.4)	476 (39.6)	.69
Cerebrovascular disease, n (%)		224 (17.9)	7 (13.2)	217 (18.1)	.47
Chronic kidney disease, n (%)		212 (28.3)	11 (20.8)	212 (28.3)	.69
Ischemic heart disease, n (%)		365 (29.1)	25 (47.2)	340 (28.3)	.005
Valvular heart disease, n (%)		217 (17.3)	9 (17.0)	208 (17.3)	>.99
De novo HF^a^, n (%)		612 (48.8)	29 (54.7)	583 (48.5)	.46
Atrial fibrillation, n (%)		417 (34.7)	10 (10.9)	417 (34.7)	.03
QRS duration≥120 ms, n (%)		318 (25.4)	17 (32.1)	301 (25.1)	.32
LVEF^b^ (%), mean (SD)		35.3 (14.7)	28.5 (11.9)	35.6 (14.7)	.002
NT-proBNP^c^ (pg/mL), mean (SD)		10,373 (11,915)	17,035 (1900)	10,092 (11,879)	<.001
**QCG^d^ scores, mean (SD)**					
	QCG-Critical	0.30 (0.21)	0.57 (0.23)	0.29 (0.20)	<.001
	QCG-HF	0.65 (0.31)	0.78 (0.18)	0.64 (0.31)	<.001

^a^HF: heart failure.

^b^LVEF: left ventricular ejection fraction.

^c^NT-proBNP: N-terminal prohormone of brain natriuretic peptide.

^d^QCG: Quantitative electrocardiogram artificial intelligence system.

### Predictors of In-Hospital Cardiac Death

In the univariable logistic regression analysis, the QCG-Critical and QCG-HF scores were significant predictors of in-hospital cardiac death (Table 2). Other than QCG scores, echocardiographic LVEF, NT-proBNP level, age, ischemic heart disease, and AF were significantly correlated with in-hospital cardiac death (Table 2).

**Table 2:** Predictors of in-hospital cardiac death.

Variables		Univariate analyses			Model 1^a,b^			Model 2^b,c^	
		OR^d^ (95% CI)	*P* value	Adjusted OR (95% CI)		*P* value	Adjusted OR (95% CI)		*P* value
**QCG^e^ parameters (per 0.1 increase)**									
	QCG-Critical	1.66 (1.47-1.87)	<.001	1.68 (1.47-1.92)		<.001	1.59 (1.36-1.87)		<.001
	QCG-HF^f^	1.21 (1.08-1.37)	.001	1.22 (1.08-1.39)		.002	1.02 (0.84-1.24)		.82
LVEF^g^ (per 5% decrease)		1.21 (1.07-1.37)	.002	1.26 (1.09-1.45)		.001	*1.29 (1.10-1.51)*		*.02*
NT-proBNP^h^ (per 1000 pg/ml increase)		1.03 (1.01-1.05)	<.001	1.04 (1.01-1.06)		<.001	*1.03 (1.00-1.05)*		*.002*
**Clinical and demographic factors as covariates**									
	Age	1.03 (1.00-1.05)	.03	*1.03 (1.00-1.06)*		*.03*	*1.04 (1.01-1.08)*		*.009*
	Male	1.05 (0.60-1.82)	.88	*1.21 (0.66-2.23)*		*.53*	*1.00 (0.48-2.07)*		*.99*
	Hypertension	0.68 (0.39-1.18)	.17	*0.51 (0.27-0.99)*		*.05*	*0.42 (0.20-0.90)*		*.03*
	Diabetes mellitus	1.17 (0.67-2.03)	.58	*0.72 (0.38-1.36)*		*.31*	*0.56 (0.26-1.19)*		*.13*
	Chronic kidney disease	1.22 (0.62-2.41)	.56	*1.08 (0.49-2.34)*		*.85*	*1.06 (0.41-2.74)*		*.90*
	Cerebrovascular disease	0.69 (0.31-1.55)	.37	*0.64 (0.26-1.53)*		*.31*	*0.54 (0.18-1.68)*		*.29*
	Ischemic heart disease	2.26 (1.30-3.93)	.004	*3.00 (1.51-5.97)*		*.002*	*2.54 (1.12-5.76)*		*.03*
	Valvular heart disease	0.98 (0.47-2.03)	.95	*2.00 (0.84-4.75)*		*.12*	*1.94 (0.68-5.50)*		*.21*
	ADHF^i^ (vs de novo)	0.78 (0.45-1.36)	.38	*0.54 (0.26-1.11)*		*.10*	*0.51 (0.22-1.19)*		*.12*
	Atrial fibrillation	0.44 (0.22-0.88)	.02	*0.73 (0.34-1.58)*		*.42*	*0.80 (0.32-2.00)*		*.63*
	QRS duration>120 ms	1.41 (0.78-2.55)	.25	*0.94 (0.48-1.83)*		*.85*	*1.23 (0.57-2.64)*		*.60*

^a^Adjusted for age, sex, hypertension, diabetes, chronic kidney disease, cerebrovascular disease, ischemic heart disease, valvular heart disease, heart failure type, atrial fibrillation, and QRS duration.

^b^When a variable was included as a covariate for adjustment, it was not adjusted for itself and QCG-Critical was added to the adjustment model (presented in italics).

^c^Adjusted for the same covariates as model 1 and further adjusted for left ventricular ejection fraction and N-terminal prohormone of brain natriuretic peptide.

^d^OR: odds ratio.

^e^QCG: Quantitative electrocardiogram.

^f^HF: heart failure.

^g^LVEF: left ventricular ejection fraction.

^h^NT-proBNP: N-terminal prohormone of brain natriuretic peptide.

^i^ADHF: acute decompensated heart failure.

After adjustment for age, sex, hypertension, diabetes, chronic kidney disease, cerebrovascular disease, ischemic heart disease, valvular heart disease, HF type, AF, and QRS duration, the two QCG scores remained significant predictors of in-hospital cardiac death. Moreover, the QCG-Critical score was an independent predictor of in-hospital cardiac death after further adjustment for echocardiographic LVEF and NT-proBNP level (OR 1.59, 95% CI 1.36-1.87; *P*<.001).

**Figure 2:**
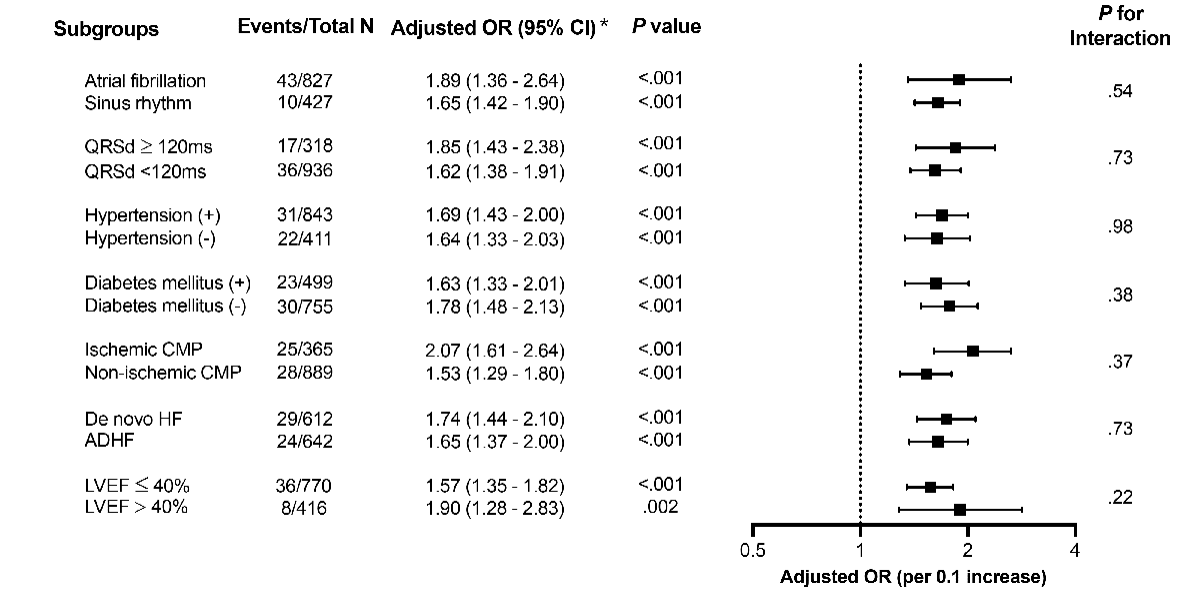
Subgroup analysis results for predicting in-hospital cardiac death. Adjusted ORs are presented for a 0.1 increase in the QCG-Critical score *Adjusted for age, sex, hypertension, diabetes, chronic kidney disease, cerebrovascular disease, ischemic heart disease, valvular heart disease, HF type, atrial fibrillation, and QRS duration (QRSd). ADHF: acute decompensated heart failure; CMP: cardiomyopathy; HF: heart failure; LVEF: left ventricular ejection fraction; OR, odds ratio.

In a subgroup analysis, the QCG-Critical score was a significant predictor of in-hospital cardiac death regardless of the initial rhythm (AF or sinus rhythm), QRS width (wide or narrow), hypertension, diabetes, HF etiology (ischemic or nonischemic), HF type (de novo or acute decompensated HF), and LVEF (HF with reduced ejection fraction vs HF with preserved or mildly reduced ejection fraction), after adjustment for other clinical parameters (Figure 2).

### QCG-Critical Score and In-Hospital Cardiac Death

**Figure 3:**
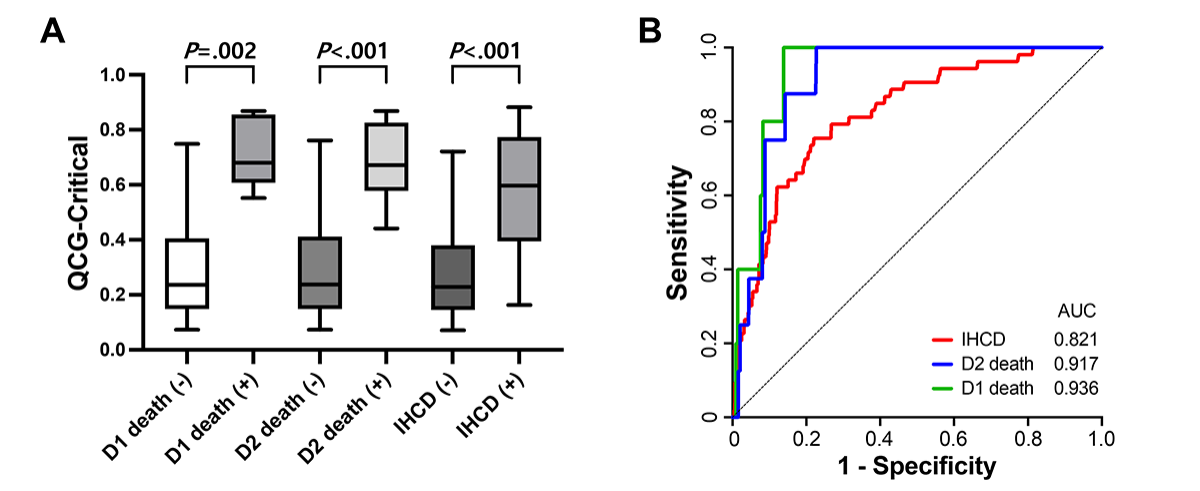
Performance of the QCG-Critical score for predicting in-hospital cardiac death (IHCD). (A) The QCG-Critical score was significantly higher in patients who experienced cardiac death within 1 day (D1), 2 days (D2), and during hospitalization than in survivors. The box-and-whisker plot is presented with 5th to 95th percentiles. (B) Performance of the QCG-Critical score presented as receiver operating characteristic curves. AUC: area under the curve; QCG: Quantitative electrocardiogram.

The QCG-Critical score was significantly higher in patients who experienced cardiac death within 1 day, 2 days, or during hospitalization than in survivors (Figure 3A). When the performance of the QCG-Critical score for predicting these events was analyzed using ROC curves, the AUC values for 1- and 2-day mortality and in-hospital cardiac death were 0.936, 0.917, and 0.821, respectively (Figure 3B).

**Figure 4:**
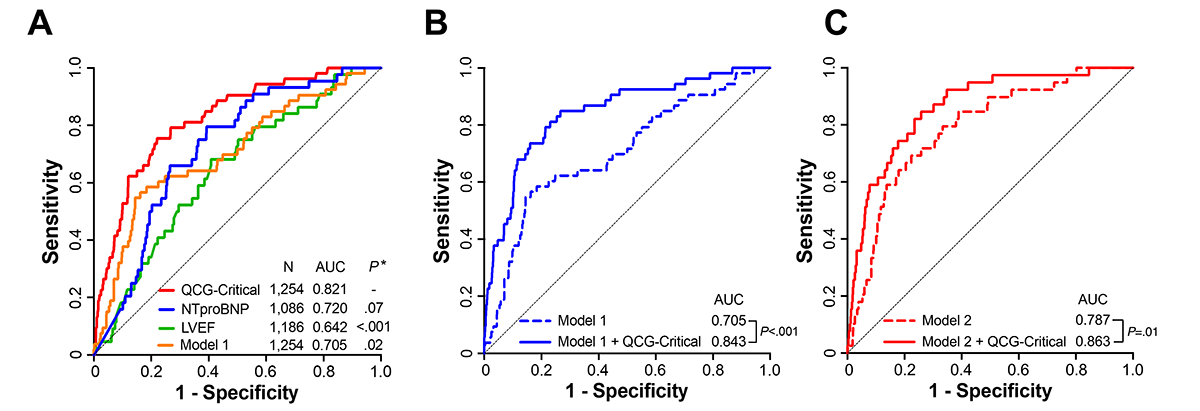
Receiver operating characteristic curves for predicting in-hospital cardiac mortality. (A) The AUC value of the QCG-Critical score was 0.821 and tended to be higher than that of echocardiographic LVEF, NT-proBNP, or a model utilizing traditional clinical variables (model 1). (B) Performance of prediction model 1 and with the addition of the QCG-Critical score. (C) Further incorporation of NT-proBNP and LVEF into the model 1 (model 2). The QCG-Critical score demonstrated additional predictive value for in-hospital cardiac mortality than model 2 alone. Model 1 includes age, sex, hypertension, diabetes, chronic kidney disease, cerebrovascular disease, ischemic heart disease, valvular heart disease, heart failure type, atrial fibrillation, and QRS duration. NT-proBNP and LVEF were further incorporated into model 2. *P value for comparison with the AUC of QCG-Critical score. AUC: area under the curve; LVEF: left ventricular ejection fraction; NT-proBNP: N-terminal prohormone of brain natriuretic peptide; QCG: Quantitative electrocardiogram.

Comparatively, the AUC values of echocardiographic LVEF and NT-proBNP level for predicting in-hospital cardiac death were 0.642 (*P*<.001 vs QCG-Critical) and 0.720 (*P*=.07 vs QCG-Critical) (Figure 4A). The AUC value of the QCG-Critical score (0.821) was significantly (*P*=.02). higher than that of model 1 (0.705) established using traditional clinical variables, including age, sex, hypertension, diabetes, chronic kidney disease, cerebrovascular disease, ischemic heart disease, valvular heart disease, HF type, AF, and QRS duration. In addition, when the QCG-Critical score was added to model 1, it significantly enhanced the prediction for in-hospital cardiac death (AUC of model 1=0.705 vs AUC of model 1 with QCG-Critical=0.843; *P*<.001) (Figure 4B). When NT-proBNP and LVEF were further included in model 1 (model 2), the QCG-Critical score again demonstrated additional predictive value for in-hospital cardiac death compared to model 2 alone (AUC of model 2=0.787 vs AUC of model 2 with QCG-Critical=0.863; *P*=.01) (Figure 4C).

### QCG-Critical Score and Long-Term Outcomes

**Figure 5:**
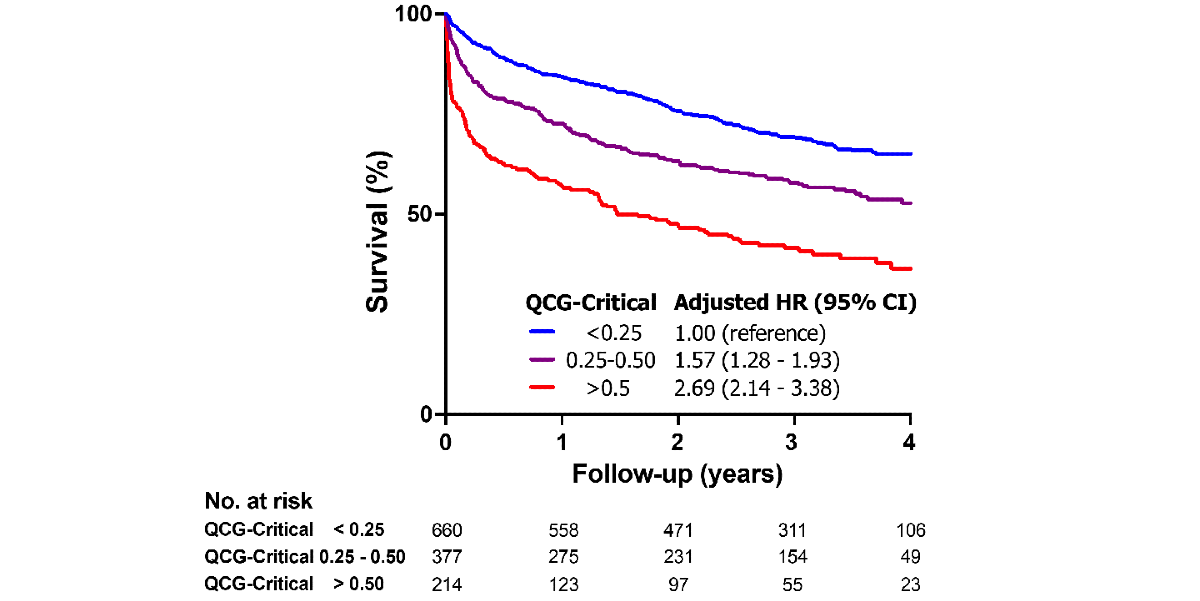
Kaplan-Meier curves for long-term mortality according to QCG-Critical scores. HR: hazard ratio; QCG: Quantitative electrocardiogram.

During a median follow-up of 2.7 years, 508 deaths occurred in the study population. To further analyze the performance of the QCG-Critical score for outcome prediction, we divided patients into three QCG-Critical score groups based on arbitrary cut-off values of 0.25 and 0.50 and then conducted survival analysis (Figure 5).

After adjustment for age, sex, comorbidities, HF etiology and type, AF, and QRS widening, patients with higher QCG-Critical scores had significantly higher all-cause mortality rates during follow-up than those with lower QCG-Critical scores (<0.25). The adjusted HRs for patients with QCG-Critical scores between 0.25 and 0.50 and for patients with QCG-Critical scores higher than 0.50 were 1.57 (95% CI 1.28-1.93) and 2.69 (95% CI 2.14-3.38), respectively (all *P*<.001). With additional adjustment for LVEF and NT-proBNP to the previous model, the adjusted HRs were 1.61 and 2.27, respectively, consistent with the main analysis (Figure S2 in the Multimedia Appendix 1).

In a subgroup analysis, a higher QCG-Critical score (>0.50 vs ≤0.50) was significantly correlated with all-cause mortality during follow-up, regardless of the initial rhythm (AF or sinus rhythm), QRS width (wide or narrow), hypertension, diabetes, HF etiology, HF type (de novo or acute decompensated HF), and LVEF (HF with reduced ejection fraction vs HF with preserved or mildly reduced ejection fraction), after adjustment for other clinical parameters (Figure S3 in Multimedia Appendix 1).

## Discussion

Predicting outcomes in patients with HF is important for guiding management and improving prognosis [15] but is often hindered by the complexity of HF pathophysiology and the presence of other comorbidities. Recently, AI algorithms based on big data from medical records have been found to be helpful in predicting the outcomes of patients with HF [16,17]; however, these algorithms are difficult to apply in daily practice and their performance requires further improvements. In this study, the QCG-Critical score, a newly developed AI-based ECG score, was well correlated with early mortality and in-hospital cardiac death during the index after adjusting for traditional clinical risk factors. Moreover, the QCG-Critical score was an independent predictor of long-term all-cause mortality in this population, suggesting that this AI-based ECG score may serve as a novel biomarker for these patients.

ECG is a cost-effective, widely available, and easy-to-perform test, and is therefore often used as a first-line evaluation for patients with cardiovascular diseases. ST-elevation myocardial infarction is a quintessential disease where ECG evaluation is critical for a timely diagnosis. Although ECG is not deterministic for an HF diagnosis, several studies have demonstrated that some ECG features are correlated with the characteristics of HF [18]. In addition, the presence of AF or QRS widening may represent ECG features reflecting unfavorable underlying hemodynamics, thus correlating with a poor prognosis [19,20]. More subtle ECG changes have also been suggested as predictors of a poor prognosis in patients with HF; however, these require high levels of experience and skill for interpretation, which may limit their applicability [21].

Theoretically, the ECG signal may contain information regarding the electric and mechanical activities of the diseased heart beyond a physician’s perception. With the assistance of AI, ECG may provide valuable information beyond its current usage. For example, Attia et al [22] reported that LVEF reduction may be detected by ECG using AI. This new application of AI-ECG was reproduced by other researchers [23,24]. In this study, the QCG-HF score also showed good performance in predicting reduced echocardiographic LVEF of less than 40%, with an AUC value of 0.884 (Figure S4 in Multimedia Appendix 1). Notably, in the above-mentioned studies, the AI-ECG–predicted LVEF was correlated with the prognosis of patients with chronic HF, whereas the AI-based ECG score had a predictive value in patients with acute HF in this study. Thus, to the best of our knowledge, this study represents an initial effort in terms of predicting the outcomes of acute HF using AI-based ECG interpretation.

The QCG-Critical score was originally trained to detect critical medical conditions that may result in shock or mortality within 1 day [12]. In this study, the QCG-Critical score predicted early cardiac mortality in patients with acute HF with high accuracy. The AUC value of the QCG-Critical score was higher than that of echocardiographic LVEF for the prediction of in-hospital cardiac death and was also higher than the AUC value of the serum NT-proBNP level, but without statistical significance. Notably, the QCG-Critical score was available for all 1254 patients enrolled in the KorAHF study, whereas LVEF and NT-proBNP results were not available in 68 (5.4%) and 168 (13.4%) patients, respectively. Considering that the KorAHF study enrolled patients from tertiary centers in Korea, a high proportion of patients with acute HF might not have the opportunity to benefit from these echocardiographic or serum biomarker tests in real-world practice. Because ECG is a widely available evaluation tool and QCG scores are derived from ECG images, the QCG-Critical score may serve as an adequate alternative biomarker for risk stratification of patients with acute HF in real-world settings with limited resources. This score may also be useful even in well-equipped centers because it would be available immediately after the ECG exam, without requiring additional waiting for echocardiography or laboratory tests. This may be beneficial for timely risk stratification in the emergency department. The QCG-Critical score was not only correlated with in-hospital cardiac death but also showed a strong association with long-term mortality. In addition, the subgroup analysis demonstrated a consistent correlation between the QCG-Critical score and clinical outcomes. These results emphasize the potential of AI-based ECG interpretation as a novel biomarker in this field.

This study has several limitations. First, the study population predominantly consisted of Asian patients; hence, further studies are needed to validate our results across different ethnicities. Second, the AI algorithm tested in this study was derived from one of the participating centers (Seoul National University Bundang Hospital). However, there was a temporal difference between patient enrollment for algorithm training (2017 to 2019) and the test population (KorAHF enrollment, 2011 to 2014), and another external center (Severance Hospital) was involved in this study. Nevertheless, this may limit the generalizability of our findings. Third, the ECG format may affect the algorithm’s performance. Although the manufacturers of the ECG devices used in the two participating hospitals differed (Philips PageWriter TC 30 and TC 70 at Seoul National University Bundang Hospital and GE Healthcare MAC 5500 and MAC VU360 at Severance Hospital), there was no significant difference in the AI algorithm performance between the hospitals. However, because the system uses printed ECG images as input, there may be problematic scenarios where the qualities of the images influence the predictive power of the biomarkers. Although some recent AI algorithm–based studies suggest further interpretation analysis, the QCG system does not support gradient-weighted class-activation mapping or similar visualization for model explainability due to the custom network architecture used. Therefore, we could not evaluate which part of the ECG images the system uses for each prediction.

In conclusion, predicting outcomes in patients with acute HF using the newly developed AI-based ECG score appears to be feasible. Thus, this score may serve as a novel biomarker for patients with HF, potentially overcoming the cost and availability constraints of current biomarkers.

## Appendix

Multimedia Appendix 1 Distribution of QCG-Critical scores (Figure S1); Kaplan-Meier curves for long-term mortality according to the QCG-Critical scores and adjusted HRs with additional adjustment for LVEF and NT-proBNP (Figure S2); subgroup analysis results for predicting long-term mortality (Figure S3); performance of the QCG-HF score for diagnosing left ventricular dysfunction (Figure S4).

## Abbreviations

AF: atrial fibrillation
AI: artificial intelligence
AUC: area under the curve
ECG: electrocardiogram
HF: heart failure
HR: hazard ratio
KorAHF: Korean Acute Heart Failure
LVEF: left ventricular ejection fraction
NT-proBNP: N-terminal prohormone of brain natriuretic peptide
OR: odds ratio
QCG: Quantitative electrocardiogram
ROC: receiver operating characteristic
