# Supplementary material for: Artificial Intelligence–Based Electrocardiographic Biomarker for Outcome Prediction in Patients With Acute Heart Failure: Prospective Cohort Study
Source: J Med Internet Res. 2024 Jul 3;26:e52139. doi: 10.2196/52139 (PMC11255523; doi:10.2196/52139)
Supplement: Multimedia Appendix 1 [file jmir_v26i1e52139_app1.docx]

**Multimedia appendix 1**

**Figure S1. Distribution of QCG-Critical score**


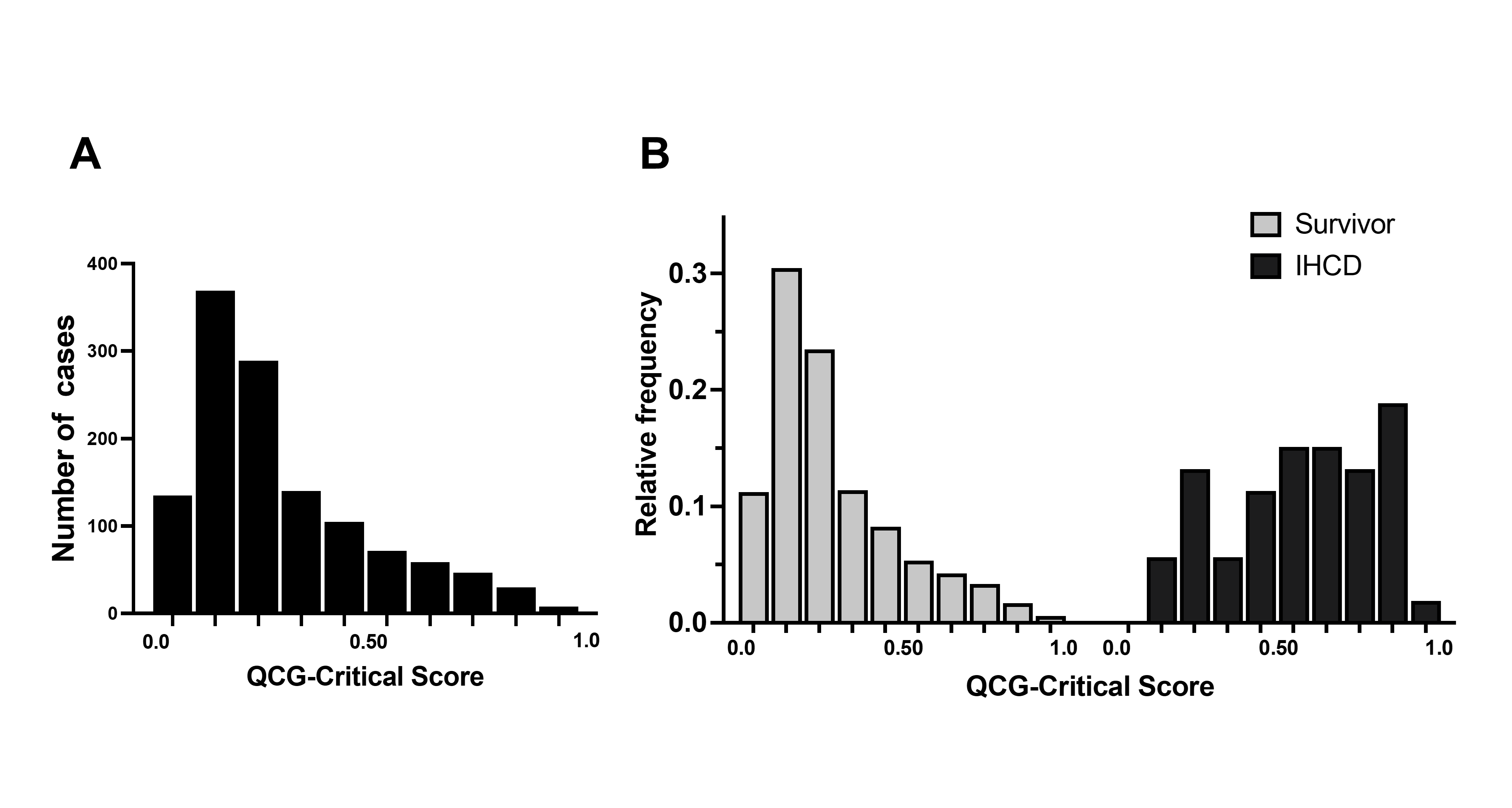


1. Distribution in total population. (B) Relative frequency according to IHCD.

IHCD, in-hospital cardiac death.

**Figure S2. Kaplan–Meier curves for long-term mortality according to the QCG-Critical scores and adjusted HR with additional adjustment for LVEF and NT-proBNP**


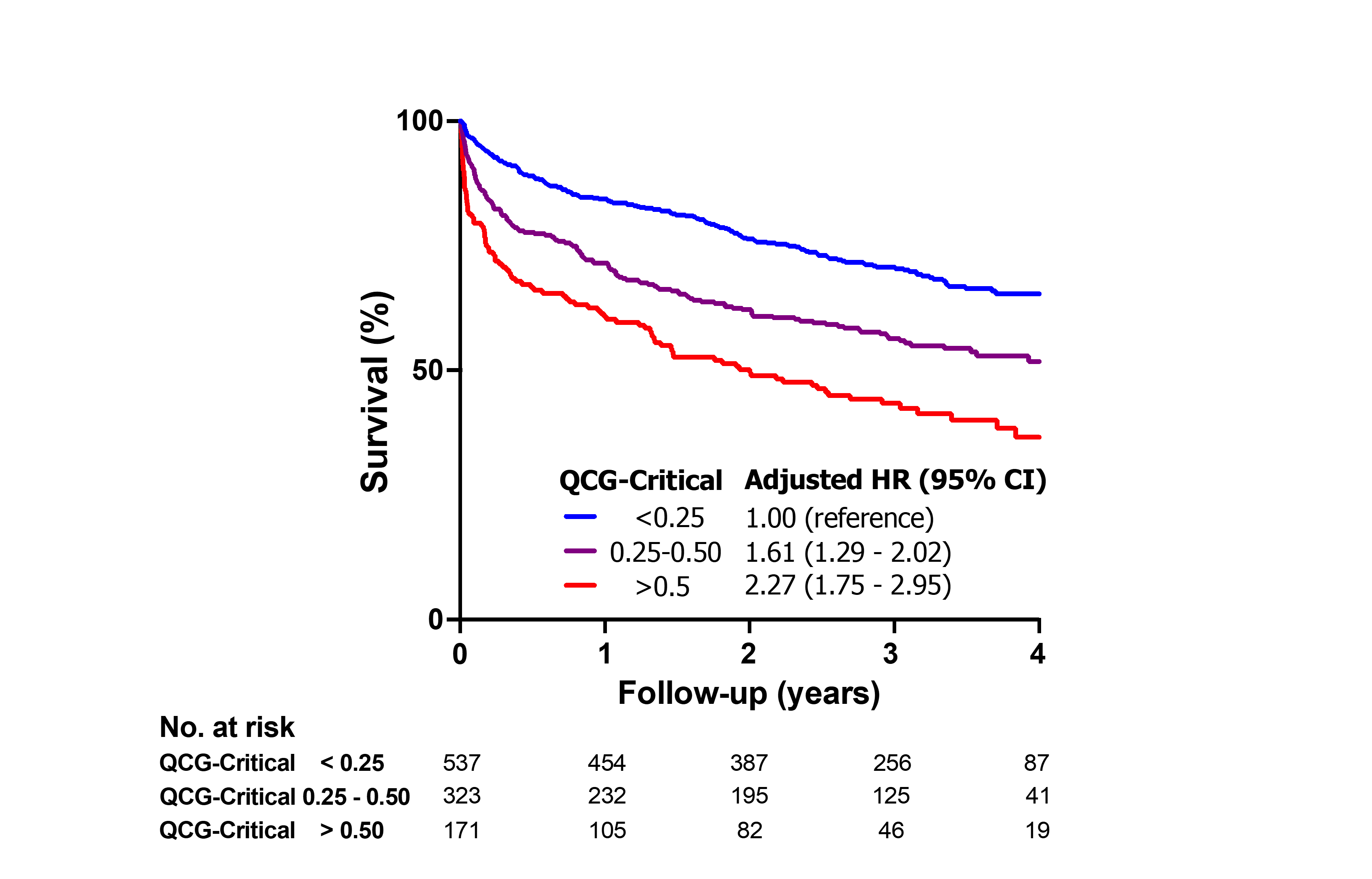


CI, confidence interval; HR, hazard ratio; LVEF, left ventricular ejection fraction; NT-proBNP, N-terminal pro-B-type natriuretic peptide.

**Figure S3. Subgroup analysis results for predicting long-term mortality**


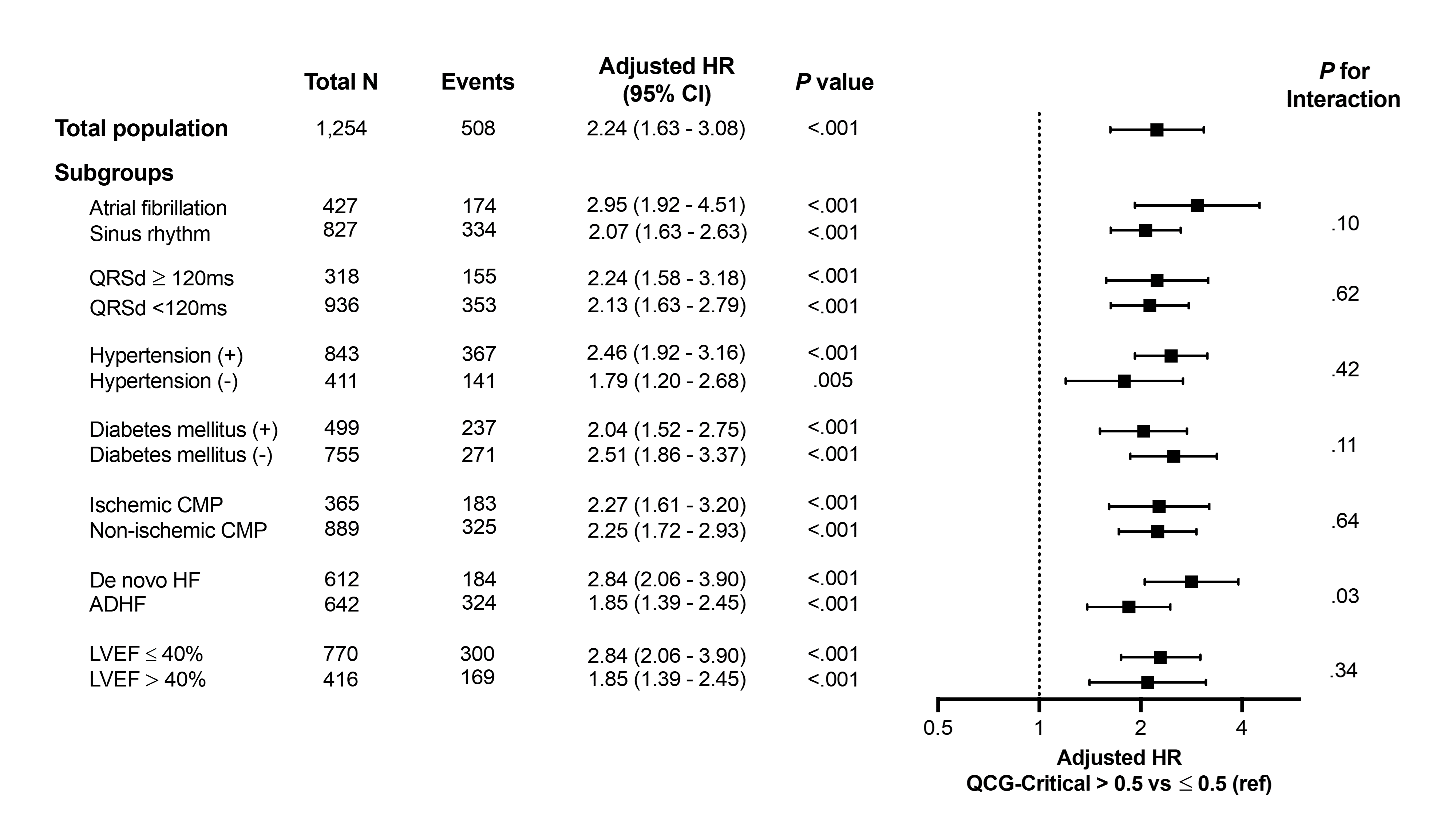


The mortality of patients with higher QCG-Critical scores (>0.50) was compared to that of patients with lower QCG-Critical scores (≤0.50).

CI, confidence interval; ADHF, acute decompensated heart failure; CI, confidence interval; CMP, cardiomyopathy; HF, heart failure; HR, hazard ratio; LVEF, left ventricular ejection fraction.

**Figure S4. Performance of the QCG-HF score for diagnosing left ventricular dysfunction**


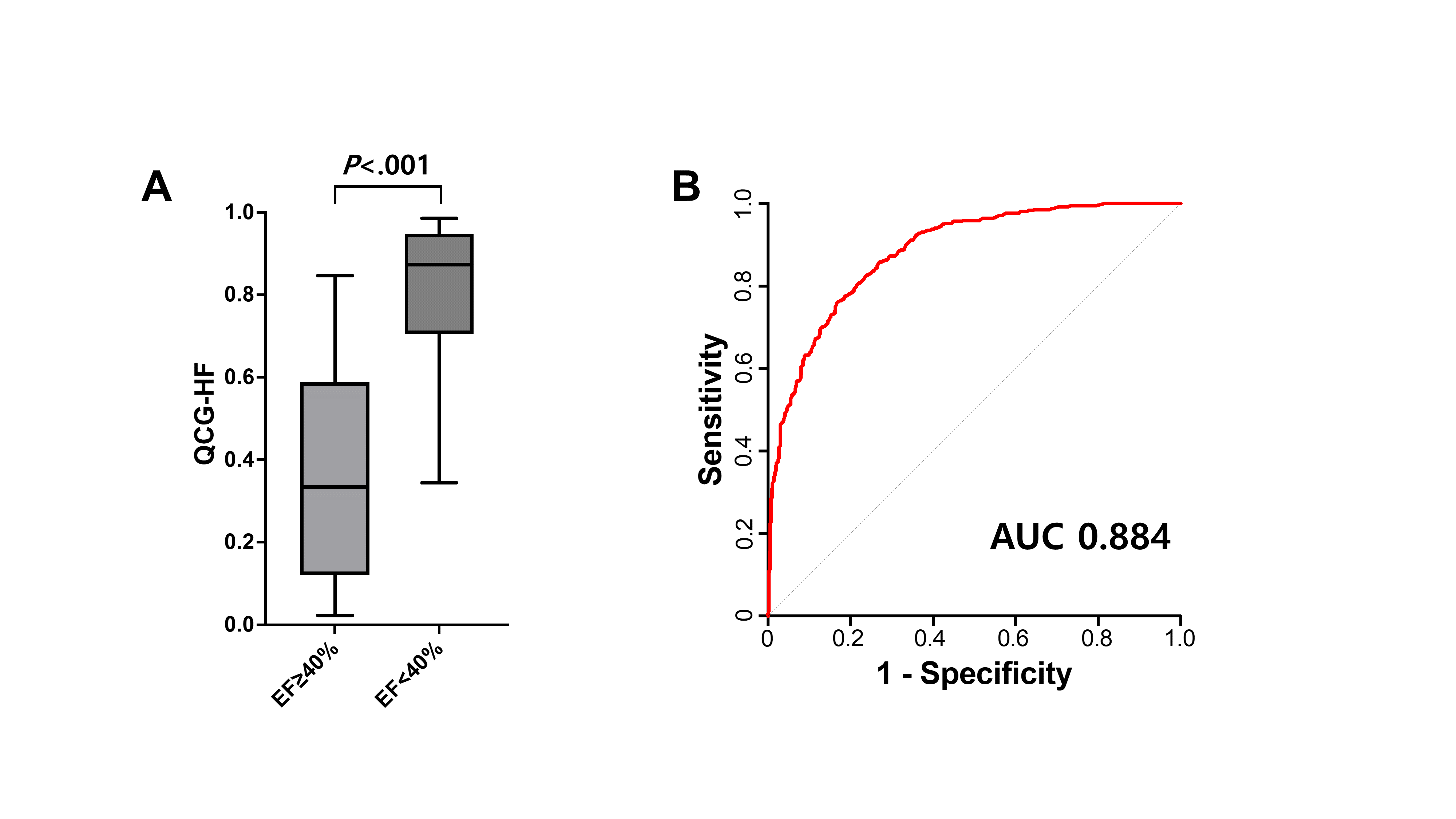


(A) The QCG-HF score was significantly higher in patients with reduced LVEF (<40%) than in those with LVEF ≥40% (0.80 ± 0.20 vs. 0.38 ± 0.27, *P*<0.001). (B) The ROC curve for predicting reduced LVEF.

AUC, area under the curve; HF, heart failure; LVEF, left ventricular ejection fraction, ROC, receiver operating characteristic.
